# Management of fever in Australian children: a population-based sample survey

**DOI:** 10.1186/s12887-020-1911-y

**Published:** 2020-01-13

**Authors:** Joanna Holt, Leslie White, Gavin R. Wheaton, Helena Williams, Shefali Jani, Gaston Arnolda, Hsuen P. Ting, Peter D. Hibbert, Jeffrey Braithwaite, on behalf of the CareTrack Kids investigative team

**Affiliations:** 1 Australian Institute of Health Innovation, Macquarie University, Level 6, 75 Talavera Rd, North Ryde, NSW 2109 Australia; 2 School of Women’s and Children’s Health, University of NSW, Sydney, Australia; 3 Sydney Children’s Hospitals Network, Westmead, Australia; 4 Division of Paediatric Medicine, Women’s and Children’s Health Network, SA Health, Adelaide, Australia; 5 Southern Adelaide Local Health Network, Adelaide, Australia; 6 Australian Centre for Precision Health School of Health Science, University of South Australia, Adelaide, Australia; 7 South Australian Health and Medical Research Institute (SAHMRI), Adelaide, Australia

**Keywords:** Fever, Children, Adherence, Guidelines

## Abstract

**Background:**

Fever in childhood is a common acute presentation requiring clinical triage to identify the few children who have serious underlying infection. Clinical practice guidelines (CPGs) have been developed to assist clinicians with this task. This study aimed to assess the proportion of care provided in accordance with CPG recommendations for the management of fever in Australian children.

**Methods:**

Clinical recommendations were extracted from five CPGs and formulated into 47 clinical indicators for use in auditing adherence. Indicators were categorised by phase of care: assessment, diagnosis and treatment. Patient records from children aged 0 to 15 years were sampled from general practices (GP), emergency departments (ED) and hospital admissions in randomly-selected health districts in Queensland, New South Wales and South Australia during 2012 and 2013. Paediatric nurses, trained to assess eligibility for indicator assessment and adherence, reviewed eligible medical records. Adherence was estimated by individual indicator, phase of care, age-group and setting.

**Results:**

The field team conducted 14,879 eligible indicator assessments for 708 visits by 550 children with fever in 58 GP, 34 ED and 28 hospital inpatient settings. For the 33 indicators with sufficient data, adherence ranged from 14.7 to 98.1%. Estimated adherence with assessment-related indicators was 51.3% (95% CI: 48.1–54.6), 77.5% (95% CI: 65.3–87.1) for diagnostic-related indicators and 72.7% (95% CI: 65.3–79.3) for treatment-related indicators. Adherence for children < 3 months of age was 73.4% (95% CI: 58.0–85.8) and 64.7% (95% CI: 57.0–71.9) for children 3–11 months of age, both significantly higher than for children aged 4–15 years (53.5%; 95% CI: 50.0–56.9). The proportion of adherent care for children attending an ED was 77.5% (95% CI: 74.2–80.6) and 76.7% (95% CI: 71.7–81.3) for children admitted to hospital, both significantly higher than for children attending a GP (40.3%; 95% CI: 34.6–46.1).

**Conclusions:**

This study reports a wide range of adherence by clinicians to 47 indicators of best practice for the management of febrile children, sampled from urban and rural regions containing 60% of the Australian paediatric population. Documented adherence was lowest for indicators related to patient assessment, for care provided in GP settings, and for children aged 4–15 years.

## Background

Fever is a common acute presentation in childhood, recently estimated to contribute 15–25% of consultations in primary care and emergency departments (EDs) [1]. Although raised body temperature has a number of causes it is most often associated with infection, the aetiology of which has continued to evolve alongside immunisation regimes [1–5].

In Australia, children with a fever may be seen by a GP, occasionally by a specialist paediatrician, or they may present to a hospital ED. A 2012 retrospective analysis found that 7.2% of febrile children under 5 years of age who presented to a specialist Australian children’s hospital had a serious bacterial infection, comprising: urinary tract infections (3.4%); pneumonia (3.4%); bacteraemia (0.4%); osteomyelitis (0.08%); meningitis (0.05%); and septic arthritis (0.04%) [6].

Most children present as mildly unwell, so the key challenge for clinicians is to quickly triage those few with serious underlying infections who are at risk of deterioration, whilst avoiding over-investigation and over-medication of the many children whose fever will resolve and only require symptomatic support and reassurance. Clinical practice guidelines (CPGs), prediction rules and pathways aim to assist clinician judgement in distinguishing self-limiting viral conditions from more serious illnesses, including life-threatening sepsis, and in managing care appropriately.

The CareTrack Kids (CTK) study retrospectively assessed care provided to a sample of Australian children aged 0–15 years, in 2012 and 2013, to determine the proportion who received care in line with CPG recommendations for 17 common conditions [7]. The proportion of care provided in accordance with CPG recommendations (adherent care) across all the 17 conditions averaged 59.8% (95% CI: 57.5–62.0) [7]. This report presents the CTK findings for children presenting with fever.

## Methods

The CTK methods have been described in detail elsewhere [7–9]. We further describe some aspects specifically relevant to fever.

### Development of indicators

A systematic search was conducted for Australian and international CPGs relating to fever in children published from 2005 to 2013. This search yielded one international guideline from the UK [10], one from a US paediatric hospital [11], one from an Australian paediatric hospital [12] and two from Australian state health entities [13, 14]. From these five CPGs, 87 recommendations were extracted and assessed for inclusion with 39 draft recommendations selected for review. During internal and external expert review, recommendations were excluded due to low acceptability, feasibility, or impact; if the concept was covered in other recommendations(s); or rated with a low appropriateness score by reviewers [9].

Thirteen recommendations were retained after review, and these were formatted into 47 medical record audit questions, hereafter referred to as ‘indicators’. Of those, 21 were restricted to specific age-groups, and 15 were restricted to specific settings (four to GPs, six to ED presentations, three to either ED presentations or inpatients, and two to either GP or ED presentations). Indicators were categorised as indicating underuse or overuse. Details of all indicators are shown in Additional file 1: Table S1.

### Sampling strategy

**Fig. 1:**
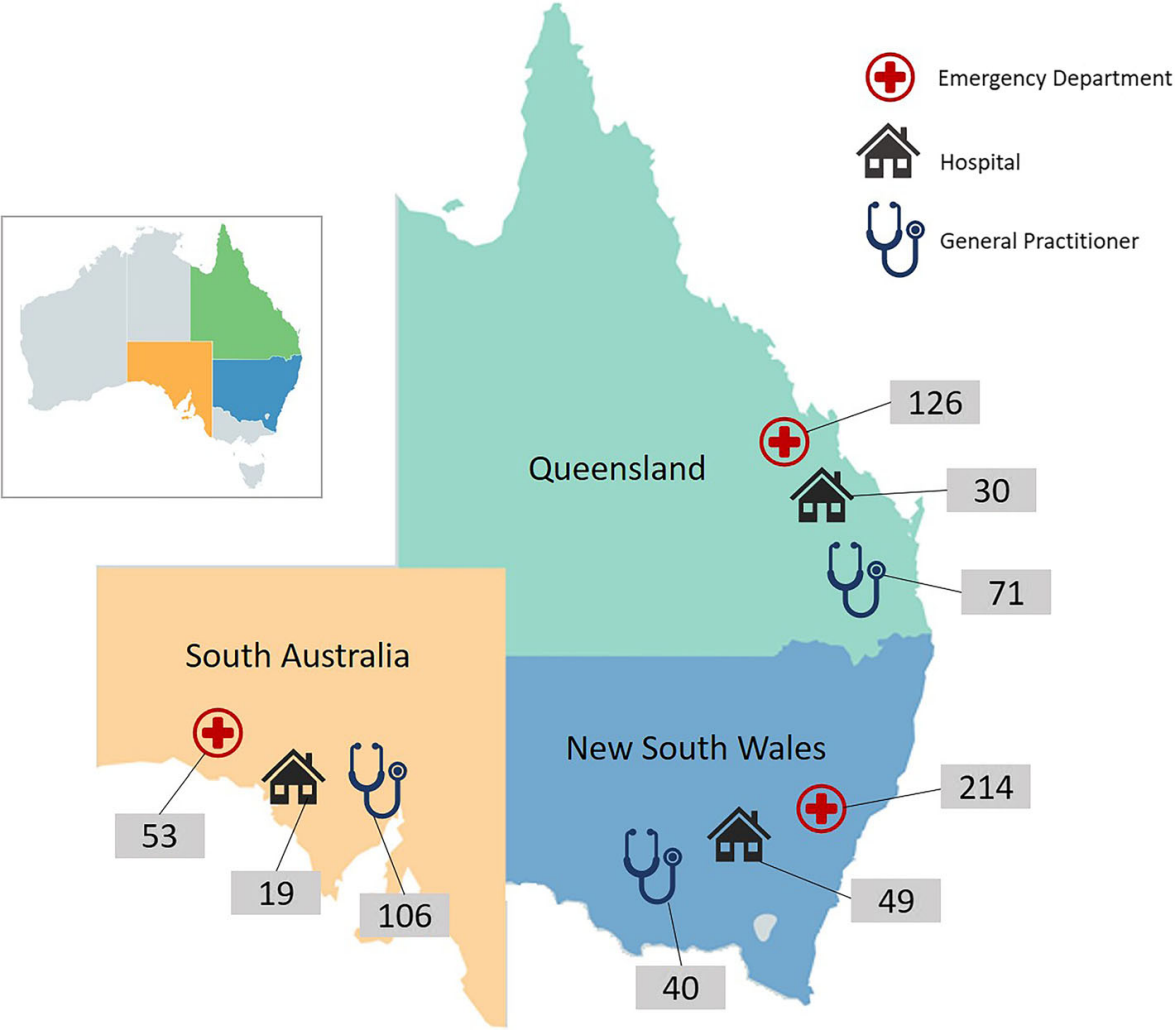
Fever assessments by state and health care provider type. Total number of visits to Emergency Departments = 393; total number of admissions to hospital = 98; total number of visits to General Practitioners = 217. Total number of fever assessments in: New South Wales = 303; Queensland = 227; and South Australia = 178. Total number of visits assessed for care of fever in sampling frame = 708. [Adapted from https://mapchart.net/, CC BY-SA 4.0]

CTK targeted 400 medical records for fever and 6000 medical records for 16 other common childhood conditions. If any of the 6400 targeted medical records contained care for fever, a separate assessment of adherent care was made for each visit. Detail on the general sampling methods are provided elsewhere [7]; additional details specific to fever can be found in Additional file 2. Briefly, four settings were sampled: hospital inpatients, ED presentations, and consultations at GPs and paediatricians’ offices in the community. These care settings were located in randomly-selected health administrative districts in Queensland, New South Wales and South Australia. Figure 1 illustrates the breakdown of assessment settings by state.

Eligible children were those aged ≤15 years who received care in 2012 and 2013. For care of fever, only one visit to a paediatrician was sampled, so this setting was removed prior to analysis. For the CTK study, the recruitment rate was 92% for hospitals, and was estimated to be 24% for GPs (Additional file 2). Nine experienced paediatric nurses, trained to assess eligibility for indicator assessment and adherence to CPGs, collected data. Medical records for selected visits in 2012 and 2013 were reviewed on-site at each participating facility during March–October 2016.

### Data analysis

Adherence was measured as the percentage of responses for each eligible indicator (i.e. answered ‘Yes’ or ‘No’) which was scored as ‘Yes’. Sampling weights were constructed as specified in Additional file 2 to adjust for oversampling of states and some settings, and for sampling within health districts [7]. The weighted data were analysed using SAS software, Version 9.4 (SAS Institute Inc., North Carolina, USA), using the SURVEYFREQ procedure. Variance was estimated by Taylor series linearization and the primary sampling unit (health district) was specified as the clustering unit. Stratification and, where appropriate, domain analyses were used (Additional file 2). Exact 95% CIs were generated using the modified Clopper-Pearson method. Results were suppressed if there were < 25 assessments.

Results were analysed for each indicator by age and grouped into phases of care relevant to the fever condition: ‘Assessment’ (documentation of relevant clinical history, signs and symptoms), ‘Diagnostics’ (any tests or investigations undertaken) and ‘Treatment’ (any therapy administered, including ongoing care and advice). Four age groups were chosen based on presumed risk differences and alignment with age group specific indicators: < 3 months; 3–11 months; 1–3 years; and 4–15 years. Phase of care results are not independent, as the same child generally has multiple phases of care in the one visit, so we report differences between point estimates but make no claims with respect to their statistical significance. Some non-contiguous age-group results (e.g., < 1 year vs > 3 years) and some settings (e.g., GPs vs hospital results) are independent and differences were compared statistically. Results were also analysed according to the setting of care.

### Ethical considerations

Primary ethics approval was received from relevant bodies including the Royal Australian College of General Practitioners (NREEC 14–008) and state hospital networks (HREC/14/SCHN/113; HREC/14/QRCH/91; HREC/14/WCHN/68), and site-specific approvals from 34 sites. All relevant bodies provided approval to waive requirements for patient consent for external access to medical records [8]. Ethics approvals included reporting by healthcare setting type for condition-level data. Participants were protected from litigation by gaining statutory immunity for CTK as a quality assurance activity, from the Federal Minister for Health under Part VC of the Health Insurance Act 1973 (Commonwealth of Australia).

## Results

### Medical records reviewed

Details of the 550 children with one or more visits for fever are provided in Table 1. Almost three-quarters of the children in the sample were under 4 years of age, with more males (56.2%) than females. Each child had 1–6 fever visits (median = 1).

**Table 1:** Characteristics of the children with fever, 2012–2013

Characteristic	Children in the CTK Study
Age^a^ - no. (%)	
< 3 months	36 (6.5)
3–11 months	104 (18.9)
1–3 years	259 (47.1)
4–15 years	151 (27.5)
Sex - no. (%)	
Male	309 (56.2)
Female	241 (43.8)

^a^The child’s age was calculated as the age at visit where there was only one, or the midpoint of the child’s age at his first and last fever visit

Of 38,023 possible indicator assessments, 13,096 (34.4%) were automatically filtered by age, setting or both, and a further 10,048 (26.4%) were assessed as not applicable or otherwise ineligible. The field team conducted 14,879 eligible indicator assessments grouped into 708 visits, at a median of 22 indicators per visit. Fever visits were assessed in 58 GP (*n* = 217), 34 ED (*n* = 393) and 28 inpatient settings (*n* = 98).

### Adherence

The estimated proportion of care adherent for each indicator is shown in Table 2. Adherence is not reported for 14 of the 47 indicators, as they had < 25 assessments. For the 33 indicators where results were reported, mean adherence was 53.5% (95% CI: 50.0–56.9) and ranged from 14.7% for indicator FEVE11 (presence of joint symptoms documented) to 98.1% for FEVE37 (infants aged < 3 months who presented to the ED had a urinalysis with culture performed). The median estimated adherence for the 33 reported indicators was 65.8% (interquartile range 39.8 to 85.1%).

**Table 2:** Adherence, by clinical indicator, 2012–2013

Indicator ID	Indicator Description	Phase of Care	No. of Children	No. of Visits	Proportion Adherent % (95% CI)
FEVE01	Children with a fever (over 38 °C) had all recent antibiotic treatment documented.	Assessment	544	699	45.9 (34.0, 58.1)
FEVE02	Neonates aged < 1 month with a fever (over 38 °C) had the GBS status of their mother documented.	Assessment	13	19	Insufficient data
FEVE03	Children with a fever (over 38 °C) had their fluid intake documented.	Assessment	548	704	53.3 (39.8, 66.4)
FEVE04	Children with a fever (over 38 °C) had their length of illness documented.	Assessment	549	707	90.4 (78.5, 97.0)
FEVE05	Children with a fever (over 38 °C) had any recent travel documented.	Assessment	548	702	16.3 (11.1, 22.8)
FEVE06	Children with a fever (over 38 °C) had their immunisation status documented.	Assessment	550	706	79.7 (66.8, 89.3)
FEVE07	Children with a fever (over 38 °C) had whether they were in direct contact with unwell people documented.	Assessment	550	706	29.0 (18.2, 42.0)
FEVE08	Children with a fever (over 38 °C) aged 4–15 years old had the presence of headaches documented.	Assessment	149	175	22.0 (5.8, 48.8)
FEVE09	Children with a fever (over 38 °C) had the presence of diarrhoea and vomiting documented.	Assessment	550	707	65.8 (59.1, 72.1)
FEVE10	Children with a fever (over 38 °C) had the presence of abdominal pain documented.	Assessment	549	705	59.3 (50.2, 67.9)
FEVE11	Children with a fever (over 38 °C) had the presence of joint symptoms documented.	Assessment	411	509	14.7 (9.5, 21.3)
FEVE12	Children with a fever (over 38 °C) had their alertness assessed.	Assessment	548	705	66.1 (56.2, 75.1)
FEVE13	Children with a fever (over 38 °C) had their vital signs assessed.	Assessment	550	708	45.9 (35.5, 56.7)
FEVE14	Children with a fever (over 38 °C) had their airway, breathing and any signs of stridor assessed.	Assessment	550	708	59.8 (44.4, 74.0)
FEVE15	Children with a fever (over 38 °C) had their circulation and capillary refill assessed.	Assessment	550	707	39.8 (32.4, 47.4)
FEVE16	Children with a fever (over 38 °C) had their cough assessed.	Assessment	549	706	61.8 (49.8, 72.9)
FEVE17	Children with a fever (over 38 °C) had their mucous membranes assessed.	Assessment	550	706	32.1 (24.4, 40.5)
FEVE18	Children with a fever (over 38 °C) aged 4–15 years old were assessed for photophobia.	Assessment	149	175	22.1 (4.2, 54.1)
FEVE19	Children with a fever (over 38 °C) were assessed for the presence of any neck stiffness.	Assessment	550	706	32.9 (23.6, 43.4)
FEVE20	Children with a fever (over 38 °C) were assessed for a rash.	Assessment	549	705	57.2 (48.3, 65.8)
FEVE21	Children with a fever (over 38 °C) were assessed for otitis media or received an examination of their eardrums.	Assessment	549	705	87.4 (74.3, 95.4)
FEVE22	Infants aged < 1 month presenting to the GP with a fever (over 38 °C) were referred to hospital.	Treatment	2	2	Insufficient data
FEVE23	Infants aged 0–3 months who presented with fever (over 38 °C) were referred to hospital.	Treatment	5	5	Insufficient data
FEVE24	Infants aged 0–3 months with a fever (over 38 °C) received a sepsis work-up.	Diagnostics	46	69	85.6 (66.1, 96.2)
FEVE25	Infants aged 0–1 months with a fever (over 38 °C) received parenteral antibiotics.	Treatment	27	42	85.1 (64.6, 96.2)
FEVE26	Children aged 3 months to 3 years with a fever (over 38 °C) who had no clear source of infection, appeared well and were fully immunised received urine microscopy.	Diagnostics	140	165	77.7 (63.9, 88.2)
FEVE27	Children aged 3 months to 3 years with a fever (over 38 °C) who had no clear source of infection, appeared well and were fully immunised were discharged home.	Treatment	133	147	92.4 (86.9, 96.1)
FEVE28	Parents of children aged 3 months to 3 years with a fever (over 38 °C) who had no clear source of infection, appeared well and were fully immunised were advised to have their child reviewed if they deteriorate.	Treatment	220	243	86.2 (73.9, 94.1)
FEVE29	Children aged ≥3 years with a fever (over 38 °C), no clinical focus and who were well were not prescribed antibiotics.	Treatment	111	126	78.8 (51.1, 95.0)
FEVE30	Infants and children who presented to ED with a fever (over 38 °C) who were shocked, unrousable OR showing signs of meningococcal disease received immediate antibiotics.	Treatment	17	17	Insufficient data
FEVE31	Infants and children who presented to ED with a fever (over 38 °C) and were shocked, unrousable OR showing signs of meningococcal disease received immediate fluid resuscitation.	Treatment	16	16	Insufficient data
FEVE32	Infants and children who presented to ED with a fever (over 38 °C) and were shocked, unrousable OR showing signs of meningococcal disease were referred or retrieved to a PICU.	Treatment	16	16	Insufficient data
FEVE33	Infants and children who presented to their GP with a fever (over 38 °C) and were shocked, unrousable OR showing signs of meningococcal disease received immediate antibiotics.	Treatment	4	4	Insufficient data
FEVE34	Infants and children who presented to their GP with a fever (over 38 °C) and were shocked, unrousable OR showing signs of meningococcal disease were transferred to hospital.	Treatment	3	3	Insufficient data
FEVE35	Infants aged < 3 months who presented to the ED with a fever (over 38 °C) had a CBE (with differential) and CRP performed.	Diagnostics	34	34	92.1 (77.5, 98.5)
FEVE36	Infants aged < 3 months who presented to the ED with a fever (over 38 °C) had blood cultures taken.	Diagnostics	34	34	96.4 (81.5, 99.9)
FEVE37	Infants aged < 3 months who presented to the ED with a fever (over 38 °C) had a urinalysis with culture performed.	Diagnostics	34	34	98.1 (86.4, 100)
FEVE38	Children with a fever (over 38 °C) who were toxic or unwell and had no focus of infection had a blood count (CBE) performed.	Diagnostics	167	215	75.2 (59.8, 87.0)
FEVE39	Children with a fever (over 38 °C) who were toxic or unwell and had no focus of infection had blood cultures taken at the same time as other blood tests.	Diagnostics	161	203	77.6 (61.8, 89.2)
FEVE40	Children aged 3 months to 3 years with a fever (over 38 °C) who showed signs of shock and had no clear source of infection had a venous blood gas taken.	Diagnostics	16	20	Insufficient data
FEVE41	Children aged 3 months to 3 years with a fever (over 38 °C) who showed signs of shock and had no clear source of infection had blood cultures taken.	Diagnostics	11	15	Insufficient data
FEVE42	Children aged 3 months to 3 years with a fever (over 38 °C) who showed signs of shock and had no clear source of infection had urine sample taken.	Diagnostics	11	15	Insufficient data
FEVE43	Children aged 3 months to 3 years with a fever (over 38 °C) who showed signs of shock and had no clear source of infection but with respiratory symptoms/signs had a chest x-ray taken.	Diagnostics	10	13	Insufficient data
FEVE44	Children aged 3 months to 3 years with a fever (over 38 °C) who showed signs of shock and had no clear source of infection were admitted to hospital for empiric IV antibiotics.	Treatment	10	10	Insufficient data
FEVE45	Children aged 3 months to 3 years with a fever (over 38 °C) who showed signs of shock and had no clear source of infection were admitted to hospital for fluid resuscitation.	Treatment	10	10	Insufficient data
FEVE46	Children with a fever (over 38 °C) where a UTI was suspected had a urine culture taken before commencing antibiotics.	Diagnostics	188	229	71.4 (36.7, 93.7)
FEVE47	Parents of children with a fever (over 38 °C) who were discharged received a fever fact sheet.	Treatment	280	322	30.6 (24.7, 37.0)

*GBS* Group B Streptococcus, *PICU* Paediatric Intensive Care Unit, *CBE* Complete Blood Examination, *CRP* C-reactive Protein, *UTI* Urinary Tract Infection

All indicators, except one, examined ‘underuse’ of recommended activities. FEVE29, the exception, measured ‘overuse’; specifically, to prescribe antibiotics for well children aged ≥3 years who had a fever with unknown clinical focus. Adherence was measured at 78.8% for this indicator (95% CI: 51.1–95.0).

**Fig. 2:**
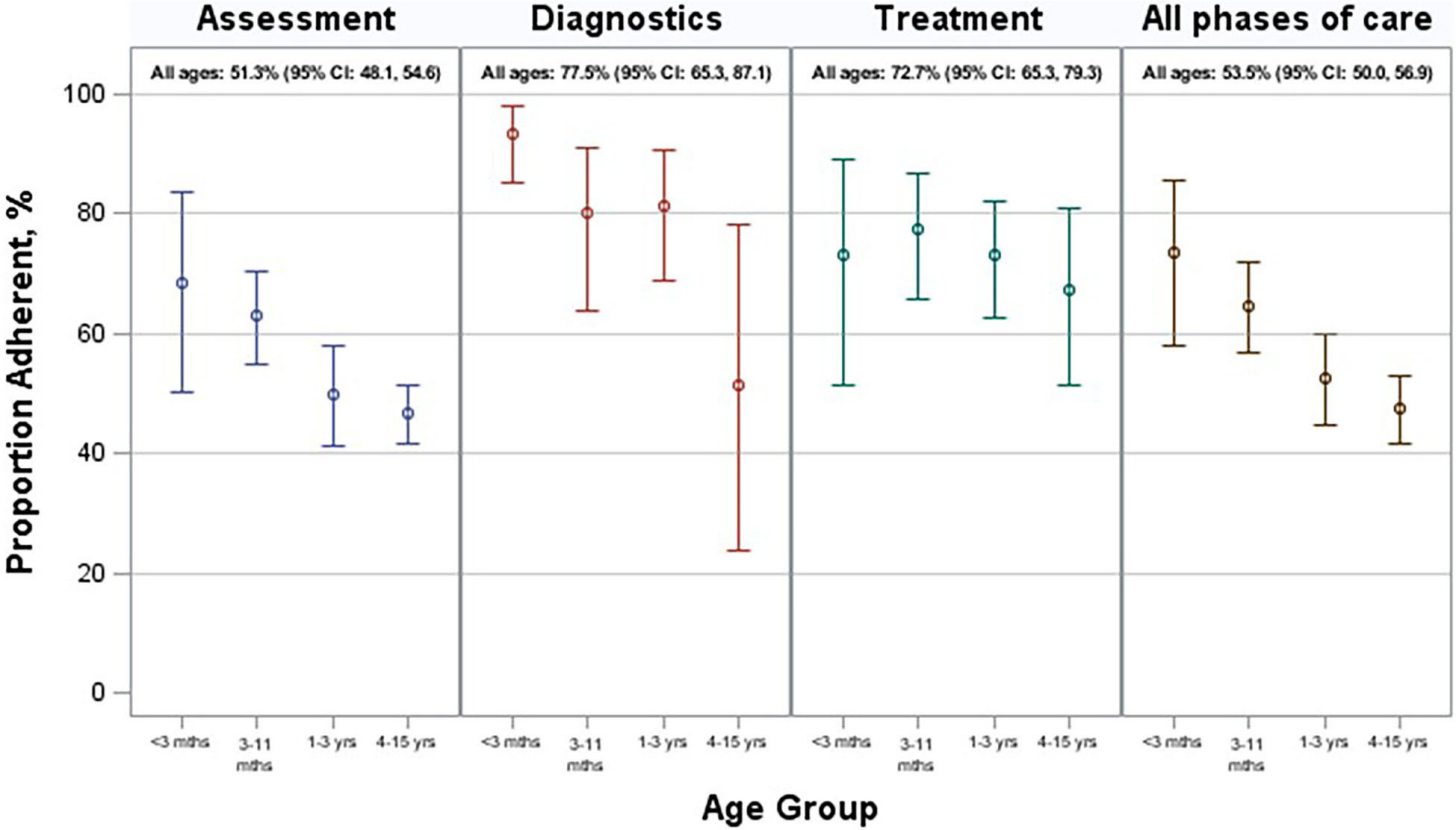
Average adherence by phase of care and age group, 2012–2013

When indicators were grouped by phase of care, adherence for Assessment-related indicators averaged 51.3% (95% CI: 48.1–54.6), over 20 percentage points lower than for Diagnostic-related indicators at 77.5% (95% CI: 65.3–87.1) and for Treatment-related indicators at 72.7% (95% CI: 65.3–79.3). Table 3 presents this information further stratified by age-group and it is graphically displayed in Fig. 2. For children under 3 months of age, adherence was 73.4% (95% CI: 58.0–85.8) and for children aged 3–11 months of age, 64.7% (95% CI: 57.0–71.9), both significantly higher than for children aged 4–15 years (53.5%; 95% CI: 50.0–56.9).

**Table 3:** Average adherence by phase of care and age group, 2012–2013

Phase of care	Age group	No. of children^a^	No. of visits	No. of indicators assessed	Proportion adherent, % (95% CI)
Assessment	< 3 months	41	64	1106	68.6 (50.3, 83.5)
	3–11 months	107	128	2173	63.0 (55.0, 70.5)
	1–3 years	265	338	6058	49.7 (41.4, 58.1)
	4–15 years	151	178	3533	46.5 (41.6, 51.5)
	Overall	550	708	12,870	51.3 (48.1, 54.6)
Diagnosis	< 3 months	40	61	248	93.5 (85.0, 98.0)
	3–11 months	85	102	188	80.0 (63.8, 91.2)
	1–3 years	179	227	433	81.4 (68.8, 90.6)
	4–15 years	75	89	177	51.2 (23.7, 78.2)
	Overall	367	479	1046	77.5 (65.3, 87.1)
Treatment	< 3 months	38	55	88	73.1 (51.2, 89.0)
	3–11 months	92	100	196	77.5 (65.8, 86.7)
	1–3 years	219	257	503	73.2 (62.5, 82.2)
	4–15 years	109	126	176	67.5 (51.5, 80.9)
	Overall	446	538	963	72.7 (65.3, 79.3)
All phases	< 3 months	41	64	1442	73.4 (58.0, 85.5)
	3–11 months	107	128	2557	64.7 (57.0, 71.9)
	1–3 years	265	338	6994	52.3 (44.6, 60.0)
	4–15 years	151	178	3886	47.3 (41.7, 52.9)
	Overall	550	708	14,879	53.5 (50.0, 56.9)

^a^The total number of children is smaller than the sum of the age-groups, as a few children had visits across two or more age-groups during 2012–2013 (e.g., one visit < 3 months of age and another at 8 months of age)

The proportion of adherent care received by children attending an ED was 77.5% (95% CI: 74.2–80.6) and 76.7% (95% CI: 71.7–81.3) for children admitted to hospital, both significantly higher than for children attending a GP (40.3%; 95% CI: 34.6–46.1); see Table 4.

**Table 4:** Average adherence by setting, 2012–2013

Healthcare setting	No. of children^a^	No. of visits	No. of indicators assessed	Proportion adherent % (95% CI)
General Practice	198	217	4322	40.3 (34.6, 46.1)
Emergency Department	342	393	8484	77.5 (74.2, 80.6)
Inpatient	93	98	2073	76.7 (71.7, 81.3)

^a^The total number of children is smaller than the sum of the settings, as children admitted to Emergency Departments are sometimes also admitted as inpatients for treatment of the same condition

## Discussion

The CTK study was a large-scale survey sampling from 60% of Australia’s paediatric population in three states, including 550 medical record reviews for febrile children presenting to GPs and EDs of general or speciality hospitals or admitted as inpatients. The fever study, as a subset of the broader CTK report [7], examined a cohort of children (0–15 years) where care for fever was documented in their clinical record. Adherence was assessed for 47 indicators derived from CPG guidelines and considered by expert panels to be reflective of best practice. As the study was retrospective, it is not possible to determine whether lack of adherence was because the recommended care was not provided by the clinician at the point of care, or simply not documented in the medical record.

It is reassuring that higher adherence seemed to be directly proportional to the degree of risk of serious underlying disease. For example, for infants < 3 months presenting to ED with a fever (FEVE35–37), appropriateness of care relating to investigations was > 90% for all three indicators. Adherence was higher for infants < 3 months than children aged 4–15 years. Others have commented on the high risk of serious bacterial infections in neonates and infants < 3 months, as well as the relative consistency of both guidelines and practice in these age groups compared to older children [1, 15]. One explanation for the lower adherence for children aged 4–15 years may be that as CPGs are focussed on care for children who are under 5 years old or less [10–14], clinicians may be less likely to use them as a guide for older children.

We found higher adherence in inpatient and ED settings than for the GP setting. It is possible that these differences reflect the inherent contextual constraints of the GP setting. Time pressures are likely to affect documentation, particularly during the Assessment phase of care when responses are a result of yes/no questioning, and when a negative result is received. There may also be a degree of assumed risk stratification when children and babies are taken to a GP rather than an ED. GPs may not consider the guidelines as reliable or valid for their practice [16] nor may they be aware of their existence [17] as the three Australian guidelines that were used for this study were released by either Departments of Health (in New South Wales [13] and South Australia [14]) or a children’s hospital [12]. None of these were endorsed by recognised GP organisations.

Clinicians were more often adherent with indicators pertaining to the Diagnostics and Treatment phases of care than for the Assessment phase of care (Fig. 2). Considering documentation as a factor in such differences, it may be more likely to be neglected during assessment when it is not an inherent function of the care process, as distinct from ordering tests or prescribing treatment.

The Assessment phase of care incorporated almost half (*n* = 21) of all 47 clinical indicators included for the fever condition, a bias supported by the literature [1, 2, 18] where emphasis is placed on gathering as much “hands-off’ information as possible about the febrile child. The average adherence during the Assessment phase of fever care exhibited wide variability in our study, ranging from 14.7 to 90.4%. In contrast, the care provided during the Diagnostics phase of care was uniformly high across the eight reported indicators. Over three-quarters of the care provided to children in this phase of care was adherent, with infants < 3 months faring particularly well (> 90% adherence). The Treatment phase of care also yielded higher adherence though no pattern emerges when analysed by age, however it should be noted that there were insufficient data to assess the appropriateness of many of the treatment decisions for infants and for toxic children in older age groups.

### Comparison with other studies

An adherence rate of 51.4% (95% CI: 43.2–59.6) was estimated in the 148 eligible children with fever (< 18 years of age) in a USA ambulatory setting, whose care was assessed for 15 indicators [19]. While the overall adherence rate is similar, results at the indicator level were not always directly comparable. For example, in the US study urine cultures were obtained for 16.2% of children 3 to 36 months of age, whereas over 78% of children in the same age group received urine microscopy in our study. Two further studies of febrile infants attending paediatric EDs in the USA, show wide variation in adherence to recommended management for febrile neonates [20], and poor adherence to current guidelines for diagnostic evaluation, particularly for infants aged 60–90 days [15]. Both studies concluded that further research is required to understand the determinants of variability before strategies can be employed to improve adherence.

### Guidelines and rules have been developed but consistency and efficacy could improve

CPGs on the management of fever in children have been developed, assessed and revised over several decades by many expert bodies to better guide practitioners in delivering appropriate care [21]. Yet, definitive conclusions on some aspects of fever management remain contested, particularly for children > 28 days, where recommended investigations and thresholds for antibiotic administration vary considerably [22]. A recent international systematic review of guidelines for the symptomatic management of fever in children identified seven common recommendations and ten discordant recommendations–mostly concerning pharmacological approach–from amongst the seven guidelines evaluated using the Appraisal of Guidelines for Research & Evaluation AGREE II tool [21].

Clinical prediction rules and models have also been developed, to improve diagnostic performance in particular [1]. A recent study, comparing four widely used clinical prediction rules and two national guidelines, found that none had perfect diagnostic accuracy and none were considered valuable in ED settings [23]. This lack of consistency and accuracy in the recommended care of children with fever present real challenges for clinicians aiming to deliver high quality care. A computer-assisted diagnostic decision system developed in Australia [18], integrating 40 clinical variables, shows more promise to improve sensitivity and thus early treatment.

### Interventions that improve adherence

Even when clinicians are aware of the evidence and are willing to change practice accordingly, altering well established care processes can be difficult without a thorough ‘due diligence’ phase (assessment of barriers and determinants prior to implementation) and a supportive environment conducive to quality improvement [24–26]. A multifaceted, organisationally relevant approach is necessary, with educational outreach, buy-in and support of both clinicians and executives, underpinned by a systemic, real-time capacity to prompt, monitor, evaluate and feedback on practice [27–30].

Organisational culture is both a determinant and a product of standardisation of care, adherence to available guidelines and quality improvement [24]. When shared purpose, teamwork and enthusiasm to learn and improve dominate organisational culture, the introduction of standardisation and of evidence-based practice finds fertile ground and far fewer obstacles [31–35].

### Strengths and weakness of the study

There are strengths and limitations to both the overall CTK study [7] and the fever-specific results reported here. Predictably, few febrile children presented directly to specialist paediatrician’s offices, requiring this setting to be removed prior to analysis. This reflects the referral pathways that are in place in Australia where a GP referral is required before a child can be seen in an ambulatory setting by a specialist paediatrician, subsidised by universal insurance.

While hospitals had excellent participation rates, we estimate that around a quarter of GPs were recruited. Accordingly, the potential impact of self-selection bias cannot be excluded, and it may have led to over-estimating adherence.

There were insufficient data to draw any conclusions about the care of neonates, infants and children in the highest risk categories (in shock, unrousable, toxic or showing signs of meningococcal disease), to come to any conclusions on the appropriateness of care for each of these important sub-cohorts. A larger sample size, or a sampling strategy targeting higher risk children, may have overcome this obstacle.

The study assessed processes of care during a visit without distinguishing between primary and subsequent visits for the same febrile episode. The study is therefore unable to provide information on issues such as the frequency of re-visits which may have resulted from missed diagnosis.

Like other studies on appropriateness of care [36, 37], the CTK study utilised medical record review to assess adherence to best practice. Clinicians may, understandably, be more inclined to document aspects of a history that are abnormal or elicit a result of positive value in elucidating the source of fever. We speculate that this may contribute to the lower levels of adherence in the GP setting as well as for the Assessment phase across all provider types. To partially mitigate this weakness, any indicators that the expert panels perceived to be unlikely to be documented were eliminated from the fever set during indicator selection. It is also possible that the opposite may have occurred, and assessments, investigations or treatments were documented without being carried out.

A strength of the study is that it did not restrict the assessment of appropriateness to just one meritorious guideline on fever. Rather, it aimed to assess best practice by selecting common recommendations from a range of reputable guidelines likely to be used by Australian clinicians. Expert groups then validated their inclusion based on acceptability, feasibility, and impact. A further strength of this study was the inclusion of all age ranges and care settings relevant to febrile illness in children.

## Conclusions

This study estimated a wide range of documented adherence to 33 indicators for the care of fever by clinicians in Australia. Overall, just over half of the care provided to children with fever was adherent, suggesting the need to further elucidate the reasons why clinicians deviate from best practice.

There are clearly opportunities for improvement, particularly in relation to the documentation of history and clinical assessment of children aged 12 months and over. Our findings suggest that appropriateness of care is relatively high for infants < 3 months of age, for children of all ages who are classified as unwell, and for children aged 3 months to 3 years with no clear source of infection.

Our findings reinforce the need for the adoption of nationally consistent guidelines tailored for the management of fever in childhood across all ages and all healthcare provider settings and regularly updated in line with the changing epidemiology of serious infection in children. Clinician access to such guidance must be quick, reliable and relevant to the care setting. The principle of planning globally whilst acting locally applies well.

## Supplementary information

**Additional file 1:** Characteristics, by clinical indicator.

**Additional file 2:** Additional details relating to study methods

## Data Availability

The datasets used and/or analysed during the current study are available from the corresponding author on reasonable request.

## Abbreviations

AGREE II: Appraisal of Guidelines for Research & Evaluation tool
CPG: clinical practice guideline
CTK: CareTrack Kids study
ED: emergency departments
FEVE11; FEVE37; FEVE35–37: Quality indicator identifications for fever
GP: general practices
SURVEYFREQ: name of the SAS procedure used for sample survey data
