# Supplementary material for: Management of fever in Australian children: a population-based sample survey
Source: BMC Pediatr. 2020 Jan 13;20:16. doi: 10.1186/s12887-020-1911-y (PMC6956501; doi:10.1186/s12887-020-1911-y)
Supplement: Supplementary file 1 — Additional file 1: Characteristics, by clinical indicator. [file 12887_2020_1911_MOESM1_ESM.docx]

**Additional file 1**

**Table S1: Characteristics, by clinical indicator**

|  | | | **No. of Sites** | | | |  | | |
| --- | --- | --- | --- | --- | --- | --- | --- | --- | --- |
| **Indicator ID** | **Indicator Description** | **Age Inclusion Criteria** | **GP** | **P*** | **ED** | **INPT** | **Level of Evidence^#^** | **Phase of Care****^‡^** | **Quality Type****^¥^** |
| FEVE01 | Children with a fever (over 38℃) had all recent antibiotic treatment documented. | 0 - 15 years | 58 |  | 34 | 28 | Consensus-based recommendation | Assessment | Underuse |
| FEVE02 | Neonates aged < 1 month with a fever (over 38℃) had the GBS status of their mother documented. | 0 - < 1 month | 2 |  | 10 | 5 | Consensus-based recommendation | Assessment | Underuse |
| FEVE03 | Children with a fever (over 38℃) had their fluid intake documented. | 0 - 15 years | 58 |  | 34 | 28 | Consensus-based recommendation | Assessment | Underuse |
| FEVE04 | Children with a fever (over 38℃) had their length of illness documented. | 0 - 15 years | 58 |  | 34 | 28 | Consensus-based recommendation | Assessment | Underuse |
| FEVE05 | Children with a fever (over 38℃) had any recent travel documented. | 0 - 15 years | 58 |  | 34 | 28 | Consensus-based recommendation | Assessment | Underuse |
| FEVE06 | Children with a fever (over 38℃) had their immunisation status documented. | 0 - 15 years | 58 |  | 34 | 28 | Consensus-based recommendation | Assessment | Underuse |
| FEVE07 | Children with a fever (over 38℃) had whether they were in direct contact with unwell people documented. | 0 - 15 years | 58 |  | 34 | 28 | Consensus-based recommendation | Assessment | Underuse |
| FEVE08 | Children with a fever (over 38℃) had the presence of headaches documented. | 4 - 15 years | 37 |  | 30 | 13 | Consensus-based recommendation | Assessment | Underuse |
| FEVE09 | Children with a fever (over 38℃) had the presence of diarrhoea and vomiting documented. | 0 - 15 years | 58 |  | 34 | 28 | Consensus-based recommendation | Assessment | Underuse |
| FEVE10 | Children with a fever (over 38℃) had the presence of abdominal pain documented. | 0 - 15 years | 58 |  | 34 | 28 | Consensus-based recommendation | Assessment | Underuse |
| FEVE11 | Children with a fever (over 38℃) had the presence of joint symptoms documented. | 1 - 15 years | 52 |  | 34 | 21 | Consensus-based recommendation | Assessment | Underuse |
| FEVE12 | Children with a fever (over 38℃) had their alertness assessed. | 0 - 15 years | 58 |  | 34 | 28 | Level II | Assessment | Underuse |
| FEVE13 | Children with a fever (over 38℃) had their vital signs assessed. | 0 - 15 years | 58 |  | 34 | 28 | Consensus-based recommendation | Assessment | Underuse |
| FEVE14 | Children with a fever (over 38℃) had their airway, breathing and any signs of stridor assessed. | 0 - 15 years | 58 |  | 34 | 28 | Level II | Assessment | Underuse |
| FEVE15 | Children with a fever (over 38℃) had their circulation and capillary refill assessed. | 0 - 15 years | 58 |  | 34 | 28 | Level II | Assessment | Underuse |
| FEVE16 | Children with a fever (over 38℃) had their cough assessed. | 0 - 15 years | 58 |  | 34 | 28 | Consensus-based recommendation | Assessment | Underuse |
| FEVE17 | Children with a fever (over 38℃) had their mucous membranes assessed. | 0 - 15 years | 58 |  | 34 | 28 | Consensus-based recommendation | Assessment | Underuse |
| FEVE18 | Children with a fever (over 38℃) were assessed for photophobia. | 4 - 15 years | 37 |  | 30 | 13 | Consensus-based recommendation | Assessment | Underuse |
| FEVE19 | Children with a fever (over 38℃) were assessed for the presence of any neck stiffness. | 0 - 15 years | 58 |  | 34 | 28 | Consensus-based recommendation | Assessment | Underuse |
| FEVE20 | Children with a fever (over 38℃) were assessed for a rash. | 0 - 15 years | 58 |  | 34 | 28 | Consensus-based recommendation | Assessment | Underuse |
| FEVE21 | Children with a fever (over 38℃) were assessed for otitis media or received an examination of their eardrums. | 0 - 15 years | 58 |  | 34 | 28 | Consensus-based recommendation | Assessment | Underuse |
| FEVE22 | Infants aged < 1 month presenting to the GP with a fever (over 38℃) were referred to hospital. | 0 - < 1 month | 2 | NA | NA | NA | Consensus-based recommendation | Treatment | Underuse |
| FEVE23 | Infants aged 0-3 months who presented with fever (over 38℃) were referred to hospital. | 0 - 3 months | 5 | NA | NA | NA | Consensus-based recommendation | Treatment | Underuse |
| FEVE24 | Infants aged 0-3 months with a fever (over 38℃) received a sepsis work-up. | 0 - 3 months | 4 |  | 21 | 13 | Consensus-based recommendation | Diagnostics | Underuse |
| FEVE25 | Infants aged 0-1 months with a fever (over 38℃) received parental antibiotics. | 0 - 1 months | 2 |  | 15 | 10 | Consensus-based recommendation | Treatment | Underuse |
| FEVE26 | Children aged 3 months to 3 years with a fever (over 38℃) who had no clear source of infection, appeared well and were fully immunised received urine microscopy. | 3 months - 3 years | NA | NA | 31 | 13 | Consensus-based recommendation | Diagnostics | Underuse |
| FEVE27 | Children aged 3 months to 3 years with a fever (over 38℃) who had no clear source of infection, appeared well and were fully immunised were discharged home. | 3 months - 3 years | NA | NA | 31 | 10 | Consensus-based recommendation | Treatment | Underuse |
| FEVE28 | Parents of children aged 3 months to 3 years with a fever (over 38℃) who had no clear source of infection, appeared well and were fully immunised were advised to have their child reviewed if they deteriorate. | 3 months - 3 years | 41 |  | 31 | 12 | Consensus-based recommendation | Treatment | Underuse |
| FEVE29 | Children aged ≥ 3 years with a fever (over 38℃), no clinical focus and who were well were not prescribed antibiotics. | 3 - 15 years | 32 |  | 27 | 7 | Consensus-based recommendation | Treatment | Overuse |
| FEVE30 | Infants and children who presented to ED with a fever (over 38℃) who were shocked, unrousable OR showing signs of meningococcal disease received immediate antibiotics. | 0 - 15 years | NA | NA | 12 | NA | Consensus-based recommendation | Treatment | Underuse |
| FEVE31 | Infants and children who presented to ED with a fever (over 38℃) and were shocked, unrousable OR showing signs of meningococcal disease received immediate fluid resuscitation. | 0 - 15 years | NA | NA | 12 | NA | Consensus-based recommendation | Treatment | Underuse |
| FEVE32 | Infants and children who presented to ED with a fever (over 38℃) and were shocked, unrousable OR showing signs of meningococcal disease were referred or retrieved to a PICU. | 0 - 15 years | NA | NA | 12 | NA | Consensus-based recommendation | Treatment | Underuse |
| FEVE33 | Infants and children who presented to their GP with a fever (over 38℃) and were shocked, unrousable OR showing signs of meningococcal disease received immediate antibiotics. | 0 - 15 years | 4 | NA | NA | NA | Consensus-based recommendation | Treatment | Underuse |
| FEVE34 | Infants and children who presented to their GP with a fever (over 38℃) and were shocked, unrousable OR showing signs of meningococcal disease were transferred to hospital. | 0 - 15 years | 3 | NA | NA | NA | Consensus-based recommendation | Treatment | Underuse |
| FEVE35 | Infants aged < 3 months who presented to the ED with a fever (over 38℃) had a CBE (with differential) and CRP performed. | 0 - 2 months | NA | NA | 20 | NA | Consensus-based recommendation | Diagnostics | Underuse |
| FEVE36 | Infants aged < 3 months who presented to the ED with a fever (over 38℃) had blood cultures taken. | 0 - 2 months | NA | NA | 20 | NA | Consensus-based recommendation | Diagnostics | Underuse |
| FEVE37 | Infants aged < 3 months who presented to the ED with a fever (over 38℃) had a urinalysis with culture performed. | 0 - 2 months | NA | NA | 20 | NA | Consensus-based recommendation | Diagnostics | Underuse |
| FEVE38 | Children with a fever (over 38℃) who were toxic or unwell and had no focus of infection had a blood count (CBE) performed. | 0 - 15 years | 16 |  | 29 | 21 | Consensus-based recommendation | Diagnostics | Underuse |
| FEVE39 | Children with a fever (over 38℃) who were toxic or unwell and had no focus of infection had blood cultures taken at the same time as other blood tests. | 0 - 15 years | 13 |  | 29 | 21 | Consensus-based recommendation | Diagnostics | Underuse |
| FEVE40 | Children aged 3 months to 3 years with a fever (over 38℃) who showed signs of shock and had no clear source of infection had a venous blood gas taken. | 3 months - 3 years | 1 |  | 11 | 4 | Consensus-based recommendation | Diagnostics | Underuse |
| FEVE41 | Children aged 3 months to 3 years with a fever (over 38℃) who showed signs of shock and had no clear source of infection had blood cultures taken. | 3 months - 3 years | 1 |  | 7 | 4 | Consensus-based recommendation | Diagnostics | Underuse |
| FEVE42 | Children aged 3 months to 3 years with a fever (over 38℃) who showed signs of shock and had no clear source of infection had urine sample taken. | 3 months - 3 years | 1 |  | 7 | 4 | Consensus-based recommendation | Diagnostics | Underuse |
| FEVE43 | Children aged 3 months to 3 years with a fever (over 38℃) who showed signs of shock and had no clear source of infection but with respiratory symptoms/signs had a chest x-ray taken. | 3 months - 3 years | 2 |  | 6 | 2 | Consensus-based recommendation | Diagnostics | Underuse |
| FEVE44 | Children aged 3 months to 3 years with a fever (over 38℃) who showed signs of shock and had no clear source of infection were admitted to hospital for empiric IV antibiotics. | 3 months - 3 years | 1 |  | 7 | NA | Consensus-based recommendation | Treatment | Underuse |
| FEVE45 | Children aged 3 months to 3 years with a fever (over 38℃) who showed signs of shock and had no clear source of infection were admitted to hospital for fluid resuscitation. | 3 months - 3 years | 1 |  | 7 | NA | Consensus-based recommendation | Treatment | Underuse |
| FEVE46 | Children with a fever (over 38℃) where a UTI was suspected had a urine culture taken before commencing antibiotics. | 0 - 15 years | 30 |  | 24 | 18 | Consensus-based recommendation | Diagnostics | Underuse |
| FEVE47 | Parents of children with a fever (over 38℃) who were discharged received a fever fact sheet. | 0 - 15 years | NA | NA | 34 | 26 | Consensus-based recommendation | Treatment | Underuse |

**Legend**: ID=Identifier; GP=General Practice; P=Paediatrician; ED=Emergency Department; INPT=Inpatient; GBS=Group B Streptococcus; PICU=Paediatric Intensive Care Unit; CBE=Complete Blood Examination; CRP=C-reactive Protein; UTI=Urinary Tract Infection.

* Paediatricians’ practices were sampled for fever, but only one record was found, so this setting was removed prior to analysis.

^#^ Level of evidence as reported in individual CPGs. CPGs used a variety of classification schemes for allocating Level of Evidence in Levels (with Level I indicating the strongest level of evidence in all classification schemes). If Levels, or a strength of recommendation in Grades, were not specified in the CPG, the term “Consensus-based recommendation” was assigned.

**^‡^** Phase of care as reported in this paper differs from that for the wider CTK study, to be more relevant to fever: Diagnosis in the wider study has been separated into Assessment and Diagnostics, while Treatment and Ongoing Management in the wider study were aggregated and reported as Treatment.

**^¥^** The type of quality of care assessed was classified as underuse or overuse: underuse refers to actions which are recommended, but not undertaken; overuse refers to actions which are not indicated, or are contraindicated, in the context of the indicator’s inclusion criteria.
