# Supplementary material for: Management of fever in Australian children: a population-based sample survey
Source: BMC Pediatr. 2020 Jan 13;20:16. doi: 10.1186/s12887-020-1911-y (PMC6956501; doi:10.1186/s12887-020-1911-y)
Supplement: Supplementary file 2 — Additional file 2: Additional details relating to study methods [file 12887_2020_1911_MOESM2_ESM.docx]

**Additional file 2: Additional details relating to study methods**

The report of top-level CareTrack Kids (CTK) study results,[1] and its associated online appendix, detail the methods which generated the data reported in this paper. Selected methods specifically relevant to fever are described below.

**Sample size**

A visit was defined as the aggregated set of condition-specific indicators as applied to an occasion of care (for inpatient care, an occasion of admitted care; for Emergency Department (ED) care, a single presentation; and for paediatricians and General Practices (GPs), a consultation). Without adjustment for the design effect, a minimum of 400 visits per condition was required to obtain overall estimates with 95% Confidence Interval (CI) and precision of +/- 5% at condition level, conservatively assuming only one eligible indicator per visit. It was anticipated that loss of precision due to design effects would be largely offset by multiple eligible indicators per visit and additional visits generated by the secondary sampling (multiple visits for care of fever for each medical record identified for sampling of fever, and visits for care of fever incidentally found in medical records identified for sampling other conditions).

**Sampling** **Process**

A multistage stratified random sampling process was implemented. For logistical efficiency, sampling was targeted at three states, Queensland (QLD), New South Wales (NSW) and South Australia (SA), which together comprise 60.0% of the Australian population aged 15 years or younger in the 2012 and 2013 calendar years. All six paediatric tertiary hospitals (two in QLD, three in NSW, and one in SA) were targeted as they have state-wide coverage. State Departments of Health organize care within health districts: Hospital and Health Services in QLD, Local Health Districts in NSW, and Local Health Networks in SA. For QLD, we targeted five health districts (two metropolitan, three regional), in NSW four health districts (two metropolitan, two regional), and in SA three health districts (two metropolitan, one regional). The sampling structure for the study is summarised in the eFigure of the previously published study results across all 17 sampled conditions.[1]

**Recruitment of healthcare providers**

Within the selected health districts, we approached all public hospitals, or private hospitals providing public services under contract, that had patient volumes of ≥2,000 ED presentations and ≥500 paediatric separations per year; we also advertised the study to GPs and paediatricians and approached all the providers we could identify through internet searches, and via personal contacts. Within the selected healthcare providers, we sampled medical records for each condition targeted at that setting. Note that 20 paediatrician’s practices were recruited and sampled for fever; only one record was found, however, so this setting was removed prior to analysis, as it could not be considered representative.

As noted in the main text, 34 of 37 (92%) eligible hospitals that were approached agreed to participate. Recruitment of GPs was decentralized. Administrative details for refusal rates, from cold-calling or direct contact by GPs who facilitated recruitment of their peers, were maintained on project laptops. At the end of recruitment all computers were decommissioned and cleaned, with the files archived on a USB. Unfortunately, the USBs created during laptop decommissioning were misplaced and have not been able to be located. This did not affect the quality indicator adherence data, as the database was remotely located and updated regularly via the internet. We have therefore sought to estimate the recruitment rate based on recruitment spreadsheets emailed to the administrative staff. For GPs, we were only able to locate emailed spreadsheets with late stage records for one state, South Australia.

Based on this spreadsheet, we approached 114 GPs and recruited 27 of them, giving a recruitment rate of 23.7%; an additional GP, not listed on the available spreadsheet, was recruited subsequently and was not added to either the numerator or the denominator, for this estimate. The spreadsheet did not have clear information on eligibility, so it is likely that an unknown number of the 114 approached were ineligible because: 1) they were not open during the whole 2012-2013 survey period; 2) they saw no or few children; or 3) they were not confident in their ability to generate full listings of children with the target conditions, or they did not use one of the four practice software systems our surveyors were trained to search. Our estimate of 23.7% is therefore likely to be an underestimate of the actual recruitment rate.

The estimated 24% recruitment rate, could lead to bias in the estimated quality of care, arising from GP self-selection. It is plausible that practices which self-selected were more confident of their adherence with quality of care indicators, potentially leading to overestimation of the quality of care in the CTK study.

**Allocation of visits to sampling units**

The number of fever records targeted at each healthcare setting was determined by a nominal allocation of the 400 records targeted, informed by data available at the time, supplemented by expert opinion, with planned over-sampling of settings where fewer occasions of care were expected.[1, 2] For hospitals, a fixed number was targeted at each site; for GPs, different combinations of conditions were targeted at each site, to simplify the logistics of sampling.

**Data collection**

Nine experienced paediatric nurses were employed across the three states, with all nine assessing occasions of care for fever. The surveyors undertook a one-week training program, prior to data collection. A surveyor manual was developed which included instructions, condition-specific definitions, inclusion and exclusion criteria, and guidance for assessing eligibility of each encounter for relevant indicators.

A web-based tool, originally developed for the CareTrack Adults study[3, 4], was designed to enter data during medical record review. Algorithms to filter indicators by healthcare setting, by age, or both, were embedded in the tool. Of the 47 fever indicators, 21 were restricted to specific age-groups, and 15 were restricted to specific settings (four to GPs, six to ED presentations, three to either ED presentations or inpatients, and two to either GP or ED presentations).

Surveyors undertook criterion-based medical record reviews using the data collection tool. Medical records for selected visits in 2012 and 2013 were reviewed on-site at each participating facility during March–October 2016. Surveyors assessed the record for evidence that the participant presented for management of fever in the years 2012 and 2013. The surveyors responded to each indicator as ‘Yes’ (care provided during the encounter was consistent with the indicator), ‘No’, or ‘Not Applicable’ (NA; the indicator was not eligible for assessment). For example, a surveyor assessing an occasion of care for a four-year old child with fever who was unwell, would record ‘NA’ to indicator FEVE29, which is restricted to children who are well.

**Analysis**

Survey or register-derived data were used to estimate the proportion of occasions of care for fever.[5-10] The number of occasions of healthcare for each condition was thereby estimated for each site (ED and inpatient) or health district (GPs), and sampling weights were calculated using the methods detailed in eAppendix 4 of the report of the top-line CTK study results (this eAppendix can be accessed by request via the corresponding author, if required).[1]

A variety of stratifications, and sometimes domain analysis,[11, 12] were necessary to ensure accuracy of the confidence interval estimates. These are detailed in eTable 2, below.

**eTable S2: Domain analysis and stratifications for different estimates presented in the manuscript.**

| Location | Sub-section/Area | Domain analysis[11, 12] | Strata |
| --- | --- | --- | --- |
| Table 2 | Indicator estimates | Yes | State and healthcare setting |
| Table 3 | Phase of care x age group estimates | Yes | Healthcare setting |
|  | Phase of care – Overall estimates | Yes | State and healthcare setting |
|  | All phases of care x age group estimates | No | Healthcare setting |
|  | All phases of care – Overall estimate* | No | State and healthcare setting |
| Table 4 | Healthcare setting estimates | No | State |

* This estimate was previously published in the top-level results for the broader CTK study.[1]

**References:**

1. Braithwaite J, Hibbert PD, Jaffe A, et al. Quality of health care for children in Australia, 2012-2013. JAMA. 2018;319(11):1113-24.

2. Hooper TD, Hibbert PD, Mealing N, et al. CareTrack Kids-part 2. Assessing the appropriateness of the healthcare delivered to Australian children: study protocol for a retrospective medical record review. BMJ Open. 2015;5(4):e007749.

3. Hunt TD, Ramanathan SA, Hannaford NA, et al. CareTrack Australia: assessing the appropriateness of adult healthcare: protocol for a retrospective medical record review. BMJ Open. 2012;2(1):e000665.

4. Runciman WB, Hunt TD, Hannaford NA, et al. CareTrack: assessing the appropriateness of health care delivery in Australia. Med J Aust. 2012;197(2):100-5.

5. Britt H, Miller GC, Henderson J, et al. General practice activity in Australia 2012-13: BEACH: Bettering the Evaluation and Care of Health. Sydney, Australia: Sydney University Press; 2013.

6. Harrison C. BEACH 2012-13 weighted data on frequency of management of selected conditions, for children aged 0-15, by General Practitioners. [Personal communication]. Menzies Centre for Health Policy, School of Public Health, The University of Sydney; 2017.

7. Australian Institute of Health and Welfare. Australian hospital statistics 2012–13: emergency department care. Health services series: Canberra, Australia: AIHW; 2013.

8. Queensland Health, New South Wales Health, South Australian Department of Health. Emergency Department data on frequency of management of selected conditions, for children aged 0-15. [Personal communication] 2017.

9. Australian Institute of Health and Welfare. Australian hospital statistics 2012–13. Canberra, Australia: AIHW; 2014.

10. Australian Institute of Health and Welfare. Inpatient separations for selected conditions, as identified by ICD-10 principal diagnoses. Retrieved from: <http://www.aihw.gov.au/hospitals-data/principal-diagnosis-data-cubes/>

11. Lohr S. Sampling: design and analysis. Second ed. Boston, MA: Brooks-Cole Publishing; 2009.

12. Heeringa SG, West BT, Berglund PA. Applied survey data analysis. Boca Raton, FL: CRC Press; 2010.
